# Inflammatory Biomarkers Aid in Diagnosis of Dementia

**DOI:** 10.3389/fnagi.2021.717344

**Published:** 2021-08-17

**Authors:** Erik B. Erhardt, John C. Adair, Janice E. Knoefel, Arvind Caprihan, Jillian Prestopnik, Jeffrey Thompson, Sasha Hobson, David Siegel, Gary A. Rosenberg

**Affiliations:** ^1^Department of Mathematics and Statistics, University of New Mexico, Albuquerque, NM, United States; ^2^Department of Neurology, University of New Mexico, Albuquerque, NM, United States; ^3^Center for Memory and Aging, Albuquerque, NM, United States; ^4^The Mind Research Network, Albuquerque, NM, United States; ^5^Department of Anesthesiology, University of New Mexico, Albuquerque, NM, United States

**Keywords:** Alzheimer's disease, vascular cognitive impairment and dementia, inflammation, diffusion tensor imaging, cerebrospinal fluid, white matter disease, machine learning

## Abstract

Dual pathology of Alzheimer's disease (AD) and vascular cognitive impairment and dementia (VCID) commonly are found together at autopsy, but mixed dementia (MX) is difficult to diagnose during life. Biological criteria to diagnose AD have been defined, but are not available for vascular disease. We used the biological criteria for AD and white matter injury based on MRI to diagnose MX. Then we measured multiple biomarkers in CSF and blood with multiplex biomarker kits for proteases, angiogenic factors, and cytokines to explore pathophysiology in each group. Finally, we used machine learning with the Random forest algorithm to select the biomarkers of maximal importance; that analysis identified three proteases, matrix metalloproteinase-10 (MMP-10), MMP-3 and MMP-1; three angiogenic factors, VEGF-C, Tie-2 and PLGF, and three cytokines interleukin-2 (IL-2), IL-6, IL-13. To confirm the clinical importance of the variables, we showed that they correlated with results of neuropsychological testing.

## Highlights

- Multimodal biomarkers facilitate biological classification of mixed dementia in cognitively impaired patients with Alzheimer's disease and vascular disease.
- Machine learning model aids in classification of this diverse group of patients by narrowing down a large number of biomarkers to those that are most important.
- Relevance of this approach is shown by correlation of those important biomarkers with neuropsychological test results.
- Proteases, angiogenic factors and cytokines in various patient groups suggest pathophysiology.

## Introduction

The need to identify patients with dementia and to determine the cause of cognitive decline during life has greatly increased as a consequence of the increase in dementia due to the aging of the world's populations. Alzheimer's disease (AD) and vascular cognitive impairment and dementia (VCID) are the major causes of dementia (Snyder et al., 2015). While the need for earlier diagnosis to facilitate treatment is generally recognized, the overlapping of symptoms, beginning in midlife, has confounded attempts at early diagnosis, promoting a search for biomarkers to aid this process (Jorgensen et al., 2020). While AD and VCID are the most common single forms of dementia, autopsy series show that mixed dementia (MX) due to dual pathologies is most common, making it important to be able to diagnose MX during life (Schneider et al., 2007; Toledo et al., 2013; Karanth et al., 2020).

Biomarkers facilitate the detection of multiple pathological processes that accumulate with aging; they provide a window on the earliest events at a time when separation of patients from effects of aging using clinical criteria alone is challenging (Sonnen et al., 2011). Biological criteria for diagnosing AD have been published in the National Institute of Aging-Alzheimer's Association (NIA-AA) research framework, which is based on the use of pathological proteins, amyloid-β (Aβ) and phosphorylated tau (pTau) in either the CSF or brain as shown by positron emission tomography (PET) along with evidence of neurodegeneration; the authors predicted that other pathological processes, such as vascular disease, could be added to the formula at a later time as new biomarkers are discovered (Jack et al., 2018). We adopted this approach to diagnose patients with MX involving dual pathology by combining white matter injury on MRI with the biological diagnosis of AD obtained from CSF. Then, to better understand the underlying pathophysiology in the expanded groups of AD, VCID, and MX, we used multiplex assays of biomarkers in CSF and blood (Craig-Schapiro et al., 2011; Pillai et al., 2019; Whelan et al., 2019; Elahi et al., 2020; Winder et al., 2020). Because of the large amount of information obtained from the multiplex assays, we used a machine-learning algorithm, Random Forests, to identify the variables of maximal importance for classifying patients into the three dementia groups. Finally, we demonstrated that the important variables had clinical relevance by correlating them with neuropsychological test results.

## Methods

### Patients and Biomarkers

The study was approved by the University of New Mexico Human Research Review Committee. All patients gave informed consent to study procedures including a lumbar puncture. Patients were recruited from neurology clinics at the University of New Mexico Hospital and the Albuquerque Veterans Administration Hospital. Patients underwent neurological examinations, neuropsychological tests, a lumbar puncture to collect CSF, a venipuncture to collect blood plasma, and a MRI. All subjects were at least 50 years old. Controls for the imaging studies were recruited from community-based volunteers. Control CSF came from patients undergoing spinal anesthesia for orthopedic surgery. ApoE genotyping was not performed.

### Cognitive Testing

Cognitive tests were administered by a trained research psychologist (JP) or trained research coordinators and scored according to standard procedures. Standardized (T) scores were calculated for each test. Averaged composite T-scores were calculated for separate cognitive domains: memory (Hopkins Verbal Learning Test-Delay, Rey Complex Figure Test-Long Delay), executive function [Digit Span Backwards, Trail Making Test B, Stroop, Controlled Oral Word Association (FAS)], attention (Digit Span Forward and Trial Making Test A), language [Boston Naming 60 item test, Controlled Oral Word Association (Animal)], and processing speed (Digit Symbol and Symbol Search, both based on WAIS-III). An overall cognitive composite score was derived as the mean of individual domain T-scores. Control participants for the MRI studies underwent the same neuropsychological test battery.

### Blood and CSF Studies

#### Phosphorylated Tau and Aβ

A number of biomarkers were measured in CSF and blood plasma. CSF biomarkers were obtained by lumbar puncture performed in the morning after fasting by one of the authors (JCA). Blood draws were performed during the same patient visit. Samples were centrifuged, aliquoted, and stored at −80°C for later analysis.

Levels of CSF Tau protein phosphorylated at threonine position 181 (pTau) were measured using the Innotest Phospho-Tau (181P) ELISA (Fujirebio US; Malvern PA). Prior to analysis, all CSF underwent one freeze-thaw cycle. Assays were performed according to manufacturer protocols and were read with a Bio-Tek multimodal plate reader with absorbance at 450 nm. The output data were used to quantify the concentrations based on the supplied in-assay standard curve. We measured β-amyloid_1−42_ (Aβ_1−42_) and β-amyloid_1−40_ (Aβ_1−40_) to calculate the Aβ_1−42_/Aβ_1−40_ ratio (V-PLEX Aβ Peptide Panel 1–6E10; MesoScale Discovery MSD, Rockville, Maryland). The output data were used to quantify the concentrations based on the 2-fold sample dilation and the supplied in-assay standard curve. All data were expressed as pg/mL, though the ratio is unitless.

#### Matrix Metalloproteinase, Angiogenesis, and Proinflammatory Assays

The biomarkers we selected were based on the MesoScale Discovery (MSD) multiplex assay kits. These have been adapted for use by the MarkVCID consortium. Matrix metalloproteinases (MMP-1, MMP-2, MMP-3, MMP-9, MMP-10) were measured with two ELISA kits (MSD; MMP 2-Plex and MMP 3-Plex). Angiogenic growth factors were measured by ELISA (MSD; Angiogenesis Panel 1). Similarly, multiple proinflammatory factors were measured with the Proinflammatory Panel 1 (MSD). For these assays, all CSF samples were run undiluted while all plasma samples were diluted 2-fold except for the MMP 3-Plex, in which case the plasma samples were diluted 10-fold. All data were expressed as pg/mL.

#### Fluid Sample Analyses

Assays were performed using established protocols on an MSD Quickplex SQ 120 plate reader, followed by analysis performed in the MSD Discovery Workbench 4.0 software that was used to quantify analyte concentrations and all data were expressed as pg/mL. Protein markers measured with MSD assays were subjected to intra-plate variability tests which calculated the coefficient of variation (CV), as determined by duplicate runs for each sample. Samples with a CV ≥ 15% were removed from further analysis. Another assessment involved two CSF and two plasma pooled control samples run in duplicate on the same plate in all assays. These control samples were held to the same intra-plate CV (≥15%) and were also assessed for plate-to-plate variability.

### MRI Studies

To obtain information on the integrity of the white matter, we used MRI scans that were performed on a Siemens 3T scanner. Initial scans were performed on a 12-channel radio frequency (RF) coil and later scans were acquired with a 32-channel RF coil. The imaging parameters with the two RF coils were closely matched. The 3D MPRAGE sequence had TR = 2530 ms, four echoes, and TI = 1200 ms with an acquisition time of 6.5 min. The 3D FLAIR sequence had a TR = 6000 ms, TE = 427 ms, and TI = 2000 ms. The diffusion data were collected with a FOV = 224, 2 mm isotropic resolution, and 72 slices for both RF coils. On the 12-channel coil, the diffusion protocol had a single-shell of b-value = 800 s/mm^2^ with 30 volumes collected with different gradient directions and five volumes with b = 0. The acquisition time was 6.5 min. The experiments done on the 32-channel coil used a CMRR multi-band sequence, which enabled us to collect more gradient directions. On the 32-channel coil, we collected three shells with a maximum b-value = 3000 s/mm^2^, 155 volumes with different gradient directions, and eight volumes with b = 0. The acquisition time was 12.5 min.

White matter hyperintensity (WMH) volume was calculated from FLAIR images based on JIM software (www.xinapse.com). The diffusion images were corrected for motion, distortion, and mean diffusivity (MD), and fractional anisotropy (FA) was calculated (www.fmrib.ox.ac.uk).

### Statistical Methods

Patient data underwent transformation, outlier detection, selection, and missing value imputation. Fluid variables measuring concentration were transformed to the log_2_ scale to mitigate right skewness; the resulting roughly symmetric distributions satisfy statistical assumptions and afford straightforward visual comparisons. Univariate outliers were identified by visual inspection and replaced with a missing value code (to be imputed later) if it was likely due to measurement error by outlying from the majority of points by roughly greater than twice the range of the majority of points on the variable's original scale. This resulted in removing roughly one or two values from about half of the features, a total of 54 values over 55 features. Observations were filtered to include patients who did not have missing values for more than 30% of the features, retaining 86 observations for our three primary diagnosis groups and controls. Missing values were imputed using the method “Multivariate Imputation by Chained Equations” via the mice R package (van Buuren and Groothuis-Oudshoorn, 2011).

Patient classification based on fluid features used Random forests (RF), a supervised ensemble machine learning algorithm that is based on classification trees (Breiman, 2001) in which many classification trees (a “forest”) are fit on bootstrapped samples of the original observations and randomly selected subsets of features. Each tree partitions the data based on a random subset of predictor variables in such a way as to obtain optimal separation between the diagnosis groups. RF provides a measure of variable importance (VIMP) for prediction accuracy, which is interpreted as the increase in prediction accuracy for decision trees within the forest with a given feature (variable) compared to decision trees without that feature; VIMP can be negative. RF also provides the marginal probability of group identity for values of each variable, and the bootstrap aggregating (bagging) technique keeps RF from overfitting. Furthermore, RF can perform multiclass prediction, automatically employs external cross-validation by predicting a patient diagnosis based on trees estimated without that patient, has minimal distributional model assumptions and is easy to implement. Variable selection improves classification and the reduced models based on classification accuracy are presented. RF was performed in R software using the package “randomForestSRC” function “rfsrc” with 10,000 trees (Ishwaran and Malley, 2014).

## Results

The three neurologists arrived at a consensus clinical diagnosis based on clinical history, neuropsychological tests and MRI FLAIR results. Initially, the results of the diffusion tensor MRI and some results of the CSF and blood studies were not available: AD CSF biomarkers were done initially, and the subsequent biomarkers in CSF and plasma were from the proteases, angiogenic factors and cytokines. Since VCID includes a number of forms of vascular disease, we focused on the small vessel form, subcortical ischemic vascular disease (SIVD), which can be detected by MRI and has a progressive course, making it more amenable to clinical trials (Pantoni, 2010). The diagnoses used were: (1) SIVD, indicating normal CSF AD proteins and abnormal white matter on FLAIR; (2) AD patients had abnormal CSF AD proteins and normal white matter; (3) MX patients had both AD proteins and white matter injury. We excluded large vessel infarcts and single strategic strokes without white matter injury. We also excluded several patients with abnormal FLAIR MRI without a cognitive deficit; they were considered white matter changes of aging.

### Demographic and Cognitive Features

Eighty-six (86) subjects had complete data permitting a full analysis; the numbers in each category are shown in Table 1. Forty-five percent of the patients were female. The median patient age was 72 years; MX patients were 7 years older than either the SIVD or AD groups (*p* = 0.010) (Table 1). Controls performed significantly better across cognitive domains than all patient groups. Memory function in the AD group was lower than in SIVD and MX (30.0 vs. 44.0 and 36.0, *p* < 0.001). There were no significant between-group differences for other cognitive domains (T-executive, T-attention, T-language, and T-processing) and composite cognitive score (T-overall).

**Table 1:** Features that are significant between diagnosis groups with Control reference values.

**Features**	**SIVD**	**Mixed**	**AD**		**Control**
**Demographics**	**(*N* = 17)**	**(*N* = 15)**	**(*N* = 19)**	***p***	**(*N* = 35)**
Age at baseline	68.0 [60.0;74.0]	76.0 [73.0;79.5]	70.0 [66.0;74.0]	0.010	65.0 [62.0;68.5]
Sex				0.039	
Female	11 (64.7%)	3 (20.0%)	9 (47.4%)		24 (68.6%)
Male	6 (35.3%)	12 (80.0%)	10 (52.6%)		11 (31.4%)
**Neuropsychological**					
T-memory	44.0 [38.0;60.5]	36.0 [25.0;41.0]	30.0 [21.0;35.5]	0.000	^**^56.0 [46.5;61.5]
**Alz disease proteins**					
A Beta 42/40 ratio^*^	7.30 [3.45;9.30]	3.30 [2.85;4.10]	4.70 [4.00;6.15]	0.010	8.70 [6.80;10.40]
P-Tau	52.0 [44.0;56.0]	97.0 [64.0;121.0]	71.0 [53.0;100.5]	0.001	54.0 [42.0;64.0]
**Protease**					
MSD CSF MMP-10	52.8 [43.1;69.2]	98.9 [91.1;119.6]	81.6 [75.6;101.9]	0.001	46.5 [34.1;67.8]
MSD Plasma MMP-3^*^	14.4 [11.7;20.7]	23.7 [17.0;34.2]	20.8 [12.0;26.7]	0.054	15.1 [11.4;21.2]
**Angiogenesis**					
CSF VEGF-C	25.3 [14.2;40.6]	25.1 [18.6;34.8]	12.7 [5.0;15.2]	0.008	19.8 [13.4;28.3]
CSF Flt-1	67.9 [60.6;87.8]	110.8 [81.9;125.4]	82.4 [67.5;93.4]	0.051	68.9 [52.2;87.7]
CSF PlGF	28.5 [16.7;41.3]	33.4 [23.9;59.7]	21.4 [18.5;23.3]	0.018	16.0 [12.1;22.0]
**Cytokine**					
CSF IL-2^*^	7.94 [6.30;9.75]	4.81 [4.50;9.63]	4.50 [4.11;7.23]	0.075	4.50 [4.50;5.95]
Plasma IL-13	0.914 [0.395;1.995]	2.529 [1.391;7.923]	1.636 [0.606;4.795]	0.043	2.552 [1.070;4.842]

*Numeric summaries are median and IQR bounds [Q1, Q3] (25th and 75th percentiles), or categorical frequencies and percentages. Three values were scaled for presentation in the table [*

* , A Beta 42/40 ratio (value*100), MSD Plasma MMP-3 (value/1000), CSF IL-2 (value*100)]. Note that the Controls with neuropsychological measurements (

** *, N = 199) were distinct from those with fluid measurements analyzed in the manuscript and are included as an external reference. P-values reported from the Kruskal-Wallis test for continuous data and from the chi-square test with continuity correction for categorical data to compare between the three diagnosis groups*.

**Figure 1:**
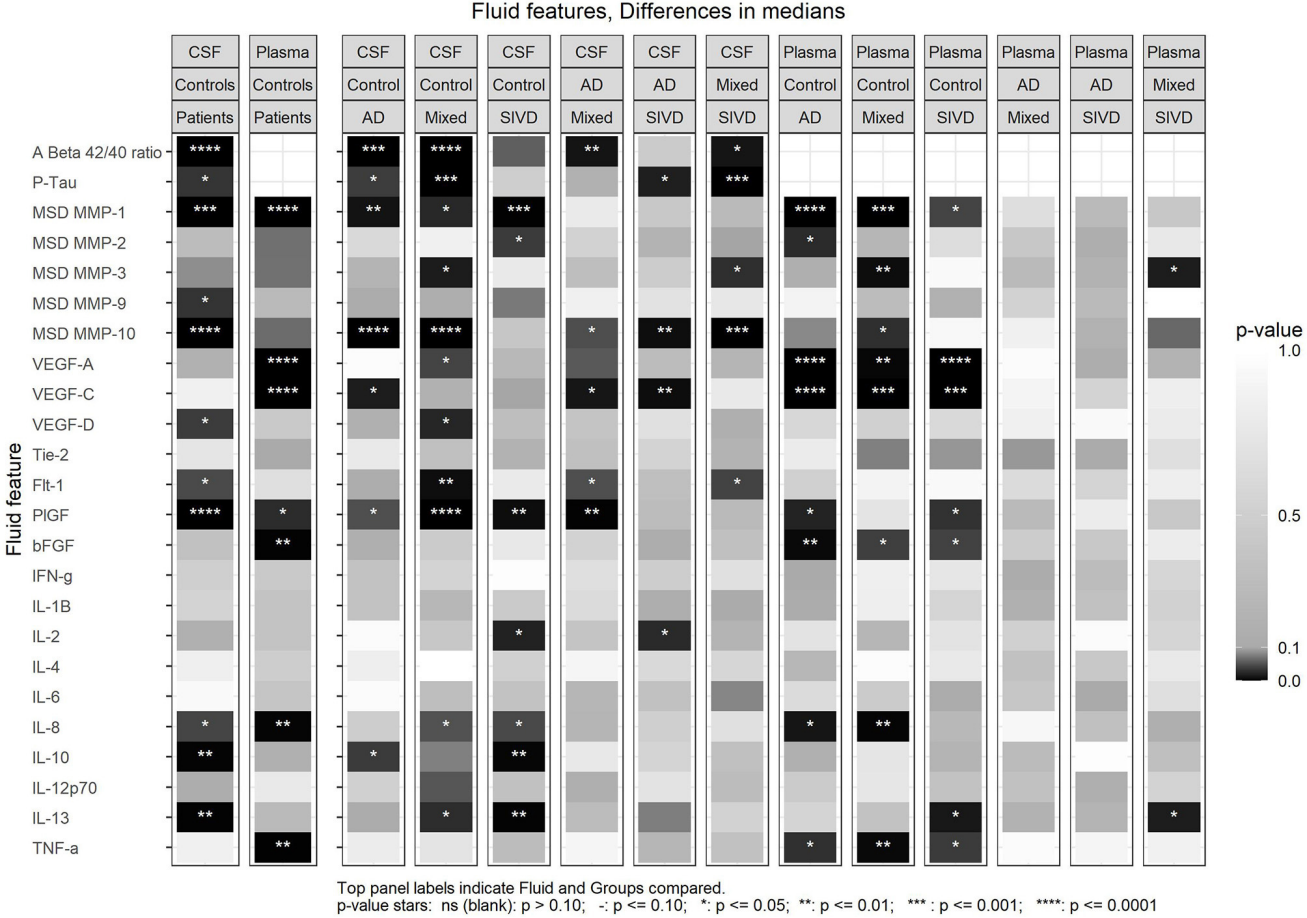
Differences in medians for all CSF and Plasma features between patient and control groups indicated by *p*-value. The first set of two panels (left) compare the combined patients groups with the control group for CSF and Plasma. The next sets of panels compare the Control group with each patient group then AD group vs. MX and SIVD and MX vs. SIVD. Some of the data is included in Table 1 and plots of all comparisons are in Supplementary Figures 1–5.

For the biomarkers, we performed several analyses. First, we compared the controls against the three patient groups combined using each of the CSF and plasma features; this showed that there were significant differences in the CSF Aβ_1−42_/Aβ_1−40_ ratio and pTau. In addition, CSF values for MMP-1, MMP-9, and MMP-10, VEGF-D, Flt-1, PlGF, IL-8, IL-10, and IL-13 were significantly different from controls (Figure 1). In plasma, MMP-1, VEGF-A, VEGF-C, PlGF, bFGF, IL-8 and TNF-α were significantly different from controls (Figure 1). Comparing controls with each patient group revealed many differences in both CSF and plasma (Figure 1). Comparing between the three groups revealed a number of significant differences between the groups in both the CSF and plasma, which tended to be much more prominent in CSF (Figure 1).

### Alzheimer's Biomarker Features

**Figure 2:**
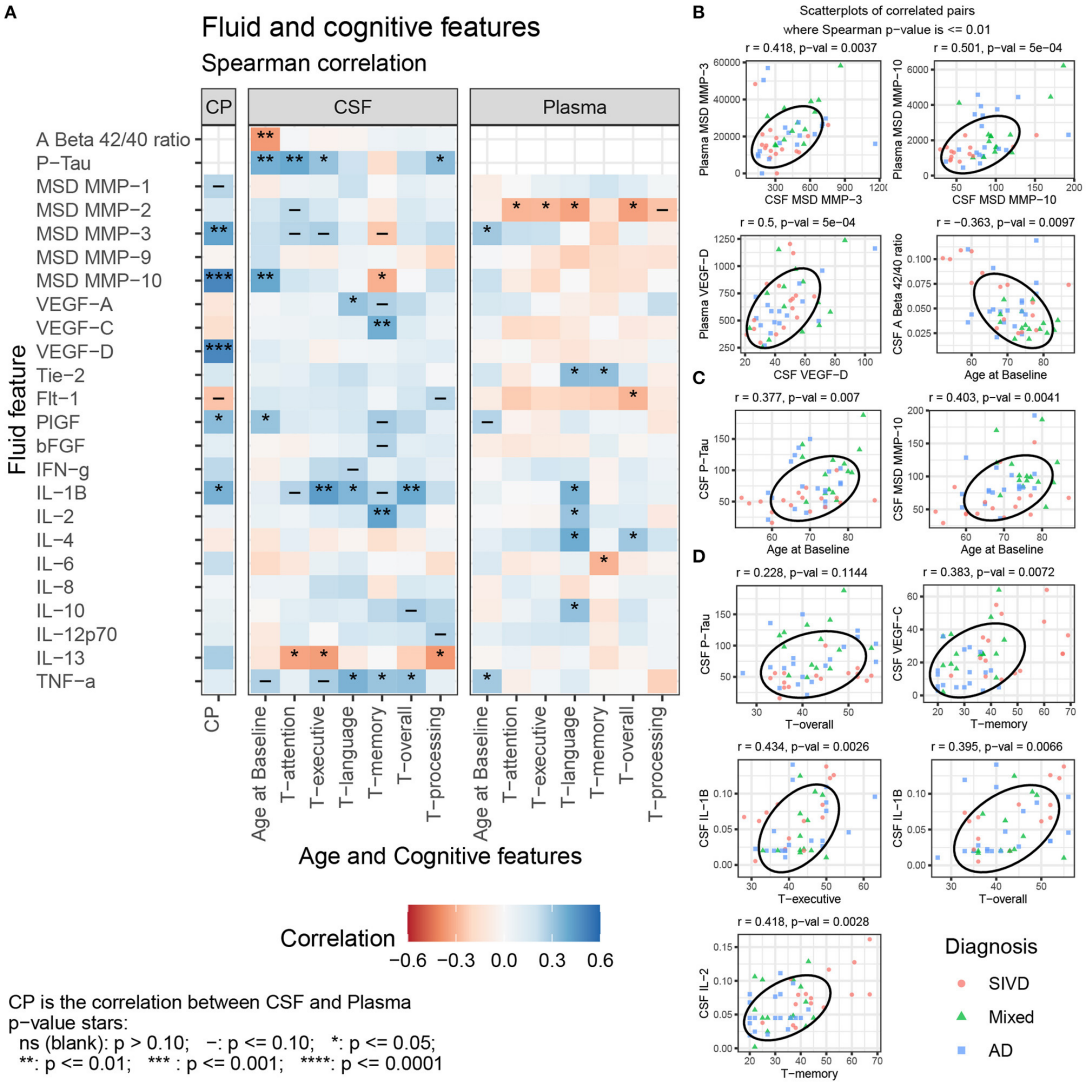
**(A)** Spearman correlation of CSF and Plasma features with Age and Cognitive features for SIVD, Mixed, and AD patients. Selected scatterplots illustrate relationships at the *p* ≤ 0.01 significance level: **(B)** same fluid features CSF vs. plasma, **(C)** fluid features with Age, and **(D)** fluid features with cognitive features.

The Aβ_1−42_/Aβ_1−40_ ratio was lower in MX than in SIVD or AD (*p* = 0.010), while pTau was higher in the MX than in SIVD or AD (*p* = 0.001) (Table 1; Supplementary Figure 2). The Aβ_1−42_/Aβ_1−40_ ratio was negatively correlated with age but not with any of the cognitive features, while pTau was positively correlated with age, attention, executive function, and processing speed (Figure 2).

### Protease Features

CSF MMP-10 was highest in MX and AD relative to SIVD (*p* = 0.001) (Table 1; Supplementary Figure 3). No other median differences between patient groups were observed, including CSF MMP-1,−2,−3, and−9, and Plasma MMP-1,−2,−9, and−10. CSF MMP-10 positively correlated with age and negatively with memory scores. There were no significant between-group differences in plasma MMPs except plasma MMP-3, which showed a trend toward significance (*p* < 0.054). Plasma MMP-2 negatively correlated with most of the cognitive measures (attention, executive function, language, and overall), and plasma MMP-3 positively correlated with age (Figure 2). CSF MMP-3 and MMP-10 correlated with plasma values for both proteases (Figure 2).

### Angiogenesis Features

CSF Placental growth factor (PlGF) was elevated in MX relative to AD (*p* = 0.018) and CSF VEGF-C was lower in AD relative to SIVD and MX (*p* = 0.008) (Table 1; Supplementary Figure 4). No other median differences between patient groups were observed in CSF for the angiogenic features VEGF-A, VEGF-D, Tie-2, Flt-1, and bFGF. In addition, there were no significant between-group differences in plasma angiogenesis features (VEGF-A, VEGF-C, VEGF-D, Tie-2, Flt-1, PlGF, and bFGF). CSF PlGF was the only angiogenesis factor correlated (positively) with age. CSF VEGF-A was positively correlated with language, and CSF VEGF-C is positively correlated with memory (Figure 2). Plasma Tie-2 is positively correlated with language and memory and Plasma Flt-1 is negatively correlated with the overall cognitive features. CSF VEGF-D and PlGF correlated with plasma values (Figure 2).

### Cytokine Features

None of the CSF cytokine features showed median differences (IFN-γ, IL-1β, IL-2, IL-4, IL-6, IL-8, IL-10, IL-12p70, IL-13, and TNF-α). Plasma IL-13 was lower in SIVD relative to MX (*p* = 0.043) (Table 1; Supplementary Figure 5). No other median differences between patient groups were observed for plasma.

CSF IL-1β was positively correlated with executive function, language, and overall cognition, CSF IL-2 was positively correlated with memory, TNF-α was positively correlated with language, memory, and overall cognition, and CSF IL-13 was negatively correlated with attention, executive function, and processing speed (Figure 2). Plasma IL-1β, IL-2, IL-4, and IL-10 were positively correlated with language, plasma IL-4 alone was positively correlated with overall cognition, while plasma IL-6 was negatively correlated with memory. Plasma TNF-α was positively correlated with age.

### Biomarker Stratification of Patients Into SIVD, MX, and AD

**Figure 3:**
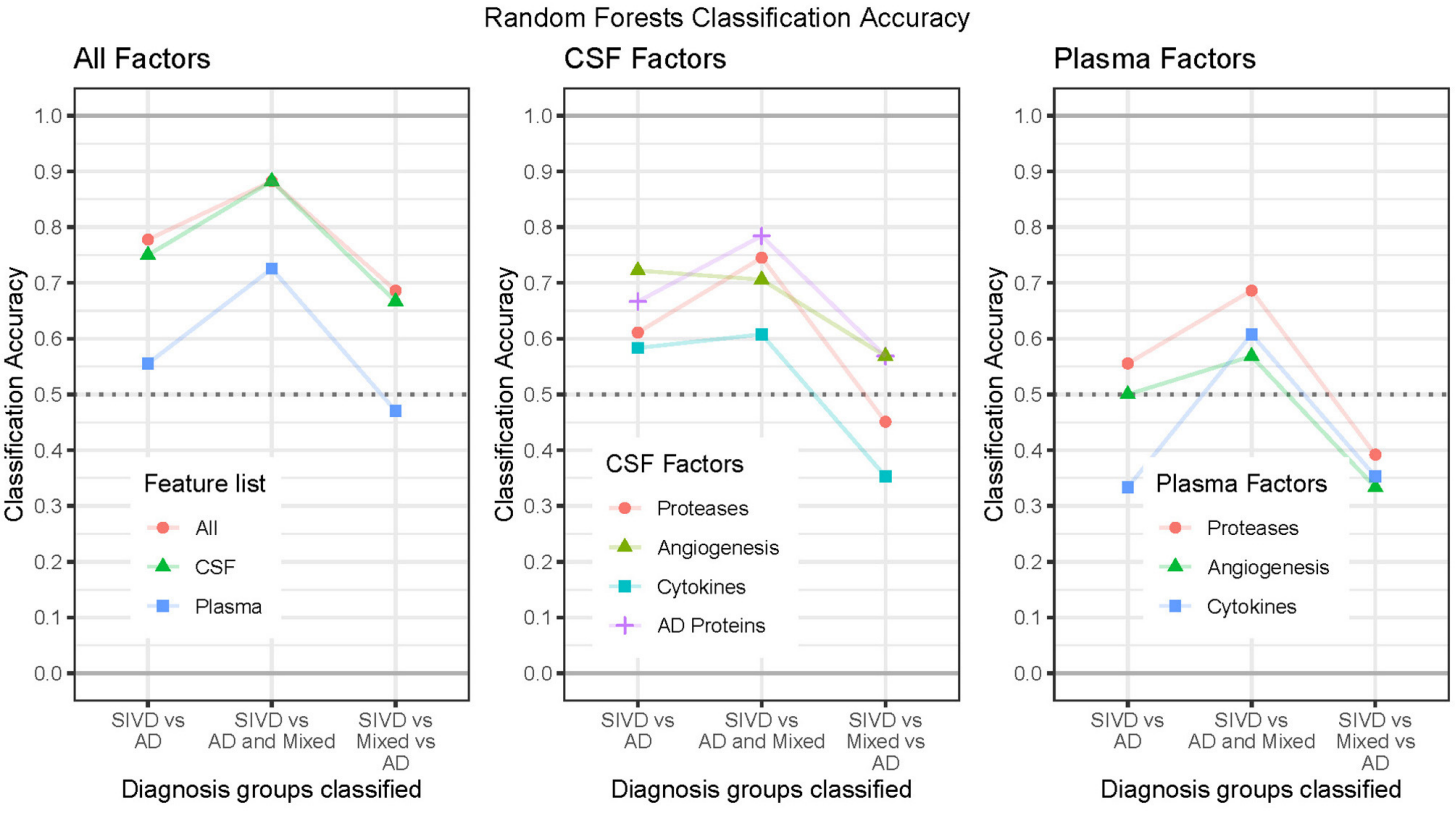
Classification accuracy by feature list considered and diagnosis grouping.

**Figure 4:**
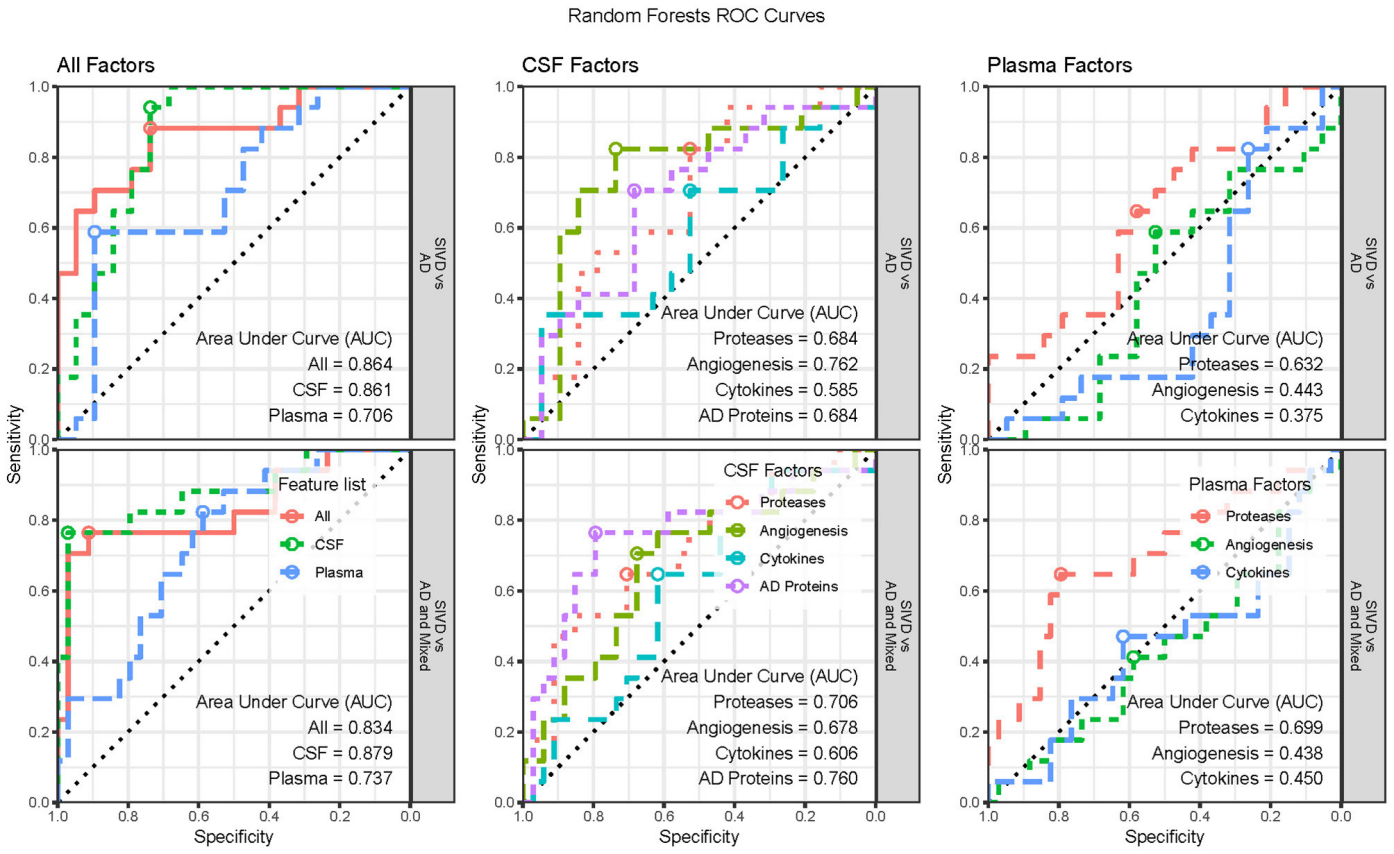
Receiver Operator Characteristic (ROC) Curves for the two-group classification scenarios by feature list and groups classified. Three-group ROC Curves are not available. The optimal threshold is indicated with a circle and the area under the curve (AUC) statistic is provided for each set of features.

We performed supervised classification using Random Forests with subsets of features from CSF and plasma to classify diagnosis groups in three ways (SIVD vs. AD, SIVD vs. AD and MX, and SIVD vs. MX vs. AD). We considered three broad scenarios. First, we considered “All Factors” of CSF and plasma together, as well as CSF and plasma features separately. Second, we considered the separate “CSF Factors” of AD Proteins, Proteases, Angiogenesis, and Cytokines. Third, we considered the separate “Plasma Factors” of Proteases, Angiogenesis, and Cytokines. To improve classification accuracy, each model is first fit using the complete set of features and then we perform manual stepwise backward selection based on variable importance (VIMP) until all remaining variables have reliably positive VIMP values. The classification accuracy results for all scenarios are summarized in Figure 3 with associated ROC curves for two-group models in Figure 4, then the variable importance values for the “All Factors” scenario are in Table 2.

**Table 2:** Variable Importance (VIMP) for important features used to classify each definition of diagnostic groups for the all factors classification scenario; cells are shaded darker for larger VIMP.

	**All**			**CSF**			**Plasma**		
**Accuracy:**	**77.8%**	**88.2%**	**68.6%**	**75.0%**	**88.2%**	**66.7%**	**55.6%**	**72.5%**	**47.1%**
**Feature**	**SIVD vs. AD**	**SIVD vs. AD and Mixed**	**SIVD vs. Mixed vs. AD**	**SIVD vs. AD**	**SIVD vs. AD and Mixed**	**SIVD vs. Mixed vs. AD**	**SIVD vs. AD**	**SIVD vs. AD and Mixed**	**SIVD vs. Mixed vs. AD**
Age at baseline			0.3%			−0.1%	2.8%	0.5%	2.6%
CSF A Beta 42/40 ratio		1.0%	1.2%		1.4%	1.4%			
CSF P-Tau	3.3%	2.9%	2.3%	4.0%	4.0%	2.8%			
CSF MSD MMP-9				0.4%					
CSF MSD MMP-10	3.4%	5.3%	4.6%	4.9%	6.8%	5.6%			
Plasma MSD MMP-1	1.4%	1.3%	0.8%				4.3%	3.0%	1.8%
Plasma MSD MMP-2	1.0%		0.5%					−0.4%	
Plasma MSD MMP-3		0.2%	0.1%					0.5%	0.7%
Plasma MSD MMP-10		0.2%					2.3%	1.2%	0.5%
CSF VEGF-A			0.3%			0.5%			
CSF VEGF-C	4.3%	0.7%	3.1%	3.4%	1.1%	3.8%			
CSF Tie-2	1.1%	0.4%	0.5%		0.6%	0.8%			
CSF PlGF	0.0%		1.8%	0.4%		1.8%			
Plasma Tie-2							5.2%	−0.3%	1.6%
Plasma bFGF									0.4%
CSF IL-1B	0.1%	0.0%	−0.1%	0.2%	0.6%	−0.1%			
CSF IL-2		0.7%	0.5%	1.6%	1.2%	0.7%			
CSF IL-12p70						0.4%			
CSF IL-13	1.6%		0.4%	0.7%		0.4%			
CSF TNF-a		0.1%			0.4%	−0.1%			
Plasma IFN-g			0.7%						
Plasma IL-6	0.6%	−0.1%	0.3%					0.0%	0.9%
Plasma IL-10									0.3%
Plasma IL-13		0.4%	0.3%					0.7%	1.5%

In the “All Factors” scenario the All features (CSF and Plasma) and CSF alone features have similar accuracies of roughly 77%, 88%, and 67% for the three diagnosis groups, while Plasma alone features had much lower accuracies (56%, 73%, and 47%). Therefore, the Plasma features do not add additional classification benefits to the CSF features (Figure 3, left; Table 2, top row). Additionally, a sensitivity analysis was performed by excluding the CSF Aβ_1−42_/Aβ_1−40_ ratio and pTau from the modeling; accuracies were similar for both All features (75%, 84%, and 70%) and CSF features (75%, 84%, 67%). The ROC Curves indicate the optimal threshold (Figure 4, circle) and the area under the curve (AUC) as an indication of the quality of the classifier, with values closer to 1 being better. For the two two-group models, the All Factor and CSF Factor models have AUC values between 0.83 and 0.88, but the Plasma Factor model has AUC values between 0.70 and 0.74 (Figure 4). Additionally, the sensitivity analysis excluding the CSF Aβ_1−42_/Aβ_1−40_ ratio and pTau from the modeling were similar, between 0.84 and 0.86.

Variable importance (VIMP) values for the “All Factors” scenario for the three diagnosis group definitions are given in Table 2. The features contributing the most to accurate classification are similar for the All features and CSF alone features, with the most important being CSF MMP-10, pTau, and VEGF-C. Less important CSF features also include CSF PlGF, Tie-2, VEGF-D, IL-2, IL-13, and IL-1β. When CSF variables are in the model, demographic features of Age and Sex actually worsen classification accuracy (negative VIMP values). The most important Plasma-only features are Plasma Tie-2, MMP-1, and MMP-10. Less important Plasma features include Age, MMP-3, IL-13, and IL-6.

In the “CSF Factors” scenario, separate models were considered for each set of features. The classification accuracy indicates that AD biomarkers and Angiogenesis factors are more predictive of diagnosis category than Proteases, with the Cytokines being the least predictive (Figures 3, 4). In the “Plasma Factors” scenario, Proteases added some predictive ability, with Angiogenesis and Cytokines providing almost no predictive ability (Figures 3, 4).

Features that did not improve classification (Table 2) because they contributed a classification of <0.3% for any diagnostic groups included Sex; Protease CSF MMP-1,−2, and−3, and Plasma MMP-9; Angiogenesis CSF VEGF-D, Flt-1, bFGF, and Plasma VEGF-A, VEGF-C, VEGF-D, Flt-1, and PlGF; and Cytokine CSF IFN-γ, IL-4, IL-6, IL-8, and IL-10, and Plasma IL-1β, IL-2, IL-4, IL-8, IL-12p70, and TNF-α.

## Discussion

Using the biological diagnosis to diagnose AD and MRI white matter injury to indicate vascular disease, we identified during life a group of cognitively impaired patients with dual pathology. Having separated patients into AD, VCID, and MX, we then used a large number of biomarkers determined in CSF and plasma with multiplex assay kits to determine the biomarkers of maximal importance. Finally, we used neuropsychological testing to validate the biomarkers identified. An important part of the study was the use of a statistical machine learning method to determine the relative importance of the biomarkers. In this manner, our study was a step in the realization of precision medicine for dementia studies.

We studied the variable importance of biomarkers in a diverse group of cognitively impaired patients classified into AD, SIVD, and MX. We included the MX group by expanding the AD biological research criteria to include a vascular factor to identify dual pathology patients (Jack et al., 2018). Commercially available multiplex assays identified proteases, angiogenic growth factors, and cytokines in CSF and plasma. A machine learning method, Random Forests, showed that the CSF variables of maximal importance, were MMP-1, MMP-3, MMP-10, VEGF-C, PlGF, IL-2, IL-6, and IL-13. By initially classifying patients into diagnostic groups, we were able to determine the levels of the biomarkers in each group, and showed that the highest values tended to be in the dual pathology patients. Our results show that the availability of multiplex assays to measure biomarkers in CSF and plasma during life provides data to compare with neuropathological studies, confirming the importance of multiple neuropathological processes in cognitively impaired patients (Toledo et al., 2013; Karanth et al., 2020).

The classes of biomarkers that we studied had inflammation and repair in common. We found that those with dual pathology had the highest values for the biomarkers, which is consistent with studies that show an acceleration of cognitive decline suspected to be due to the cumulative effects of the different pathological processes (Snowdon et al., 1997; Karanth et al., 2020). To obtain this data, we expanded the biological formula for AD to include a vascular factor, permitting the identification of patients with relatively pure AD and VCID as well as a group with dual pathology (Jack et al., 2018). Our results concur with other pathological and CSF studies that have identified proteolytic, angiogenic and inflammatory biomarkers as central features of the pathobiology of both AD and VCID (Tarkowski et al., 2002; Desai et al., 2009; Biron et al., 2011). Our results suggest that the MMPs and the angiogenic factors act together. The three MMPs that were most prominent, MMP-1, MMP-3, and MMP-10, are inducible enzymes with transcription factors, AP-1 and NF-kB, that would be important in inflammation; MMP-2, which was identified in plasma, but not CSF, is a constitutive enzyme that may have other roles (Candelario-Jalil et al., 2009).

Angiogenic factors have been identified in a number of studies in AD, but it is unclear whether they participate in injury or repair. It is possible to conceptualize a pathological scenario in which the growth of blood vessels begins with the proteolytic disruption of the extracellular matrix by one or more of the MMPs, which is analogous to vessel growth in tumors where the proteases remove pericytes and breakdown extracellular matrix proteins to prepare the vessels for sprouting under the control of angiogenic factors (Rundhaug, 2005). The angiogenic factors, VEGF, PlGF, and their receptors, Flt-1 and Tie-2, were identified: Flt-1 (elevated in CSF for MX compared to the other three groups) (Supplementary Figure 4), and Tie-2 (important in classification in plasma) (Table 2); they initiate vessel growth controlled by hypoxia-inducible factor-α under hypoxic conditions, which are present in both AD and VCID due to reduced cerebral blood flow as found in both conditions, but for different underlying mechanisms (Tomimoto, 2011; Iadecola, 2013).

Correlating biomarkers with neuropsychological testing was important in that it showed their clinical relevance. The relationship between fluid biomarkers and cognition is complex and, given modest correlations and small sample size, our data should be considered as hypothesis-generating rather than instructive. Positive correlations between cognitive performance and CSF levels of inflammatory cytokines pose a paradox if inflammation precedes injury to brain structure. Scatterplots in Figure 2 suggest that elevated CSF cytokines (e.g., CSF IL-2) and VEGF-C may differentially affect cognition by patient group. For example, higher cognitive scores in SIVD with elevated inflammatory factors might indicate they play a reparative role in this group.

Our results reveal the role of the angiogenic factors. It is interesting that Flt-1 besides being the receptor for VEGF, is a signaling factor for microglia (Ryu et al., 2009). Similarly, the proteases probably have multiple roles; high levels of MMP-10 were found in CSF and plasma, and it correlated with pTau, suggesting importance in AD by a mechanism that remains to be determined. Others have reported MMP-10 elevations in patients with AD (Stomrud et al., 2010; Craig-Schapiro et al., 2011; Whelan et al., 2019). Several of the biomarkers showed a correlation between values in the CSF and plasma, suggesting that plasma may be able to be used instead of CSF, particularly with the ultra-sensitive assay platforms (Janelidze et al., 2016).

Random Forests, a machine learning method, selected several of the cytokines as variables of importance for distinguishing patient groups, including IL-2, IL-6, and IL-13. These may influence the inflammatory response: IL-2 amplifies T_reg_ cells that are linked to chemokines, CCL1 and CCL20, which suppress astrocytosis, contributing to repair (Ito et al., 2019); in animals with traumatic brain injury, IL-13 impacts microglia by converting M1/M2 microglia into anti-inflammatory M2 phenotype (Miao et al., 2020); IL-13 is found in resilient AD patients that have reduced glial activation, increased neuronal survival, and preserved cognition (Barroeta-Espar et al., 2019).

There are several caveats with our data. First, patients were from a single center and only a subset had complete CSF/plasma and MRI datasets, reducing the numbers available for statistical analysis. Second, biomarkers selected were those available from MesoScale Discovery and had been used by the MarkVCID consortium, which included our group; other biomarkers and platforms with different biomarkers could have been used. Furthermore, the study was cross-sectional rather than longitudinal, precluding inferences about the temporal dynamics of analyte levels. A major caveat is the small sample size, which was further hindered by forming an additional MX group. However, despite the small numbers, the results were statistically significant. A follow-up study on a larger population is necessary to further validate the results of this present study.

In conclusion, we expanded the biological definition of AD by adding vascular factors, allowing the identification of patients with dual pathology prior to autopsy. Using Random Forests, a machine learning method, we have determined the major proteases, angiogenic factors, and cytokines of importance in classification in a diverse group of dementia patients. Following an initial classification into diagnostic groups, we identified the proteases, MMP-1, MMP-3 and MMP-10, the angiogenic factors, VEGF-C, PlGF, Flt-1, Tie-2, and the cytokines, IL-2, IL-6, and IL-13. Our results suggest that the combined action of proteases and angiogenic growth factors may be important in dementia with cytokines fueling the inflammatory processes. Further studies in larger numbers of patients will be needed to confirm these results.

## Data Availability Statement

The raw data supporting the conclusions of this article will be made available by the authors, without undue reservation.

## Ethics Statement

The studies involving human participants were reviewed and approved by University of New Mexico Human Research Review Committee. The patients/participants provided their written informed consent to participate in this study.

## Author Contributions

EE performed the statistical analysis and wrote a draft. JA and JK recruited the patients. AC performed the MRIs and analyzed the MRI data. JT and SH analyzed the CSF and blood. JP performed the neuropsychological testing. DS obtained the control CSF during surgery. GR obtained the funding, recruited patients, and contributed to the writing of the manuscript. All authors contributed to the article and approved the submitted version.

## Conflict of Interest

The authors declare that the research was conducted in the absence of any commercial or financial relationships that could be construed as a potential conflict of interest.

## Publisher's Note

All claims expressed in this article are solely those of the authors and do not necessarily represent those of their affiliated organizations, or those of the publisher, the editors and the reviewers. Any product that may be evaluated in this article, or claim that may be made by its manufacturer, is not guaranteed or endorsed by the publisher.

## References

[B1] Barroeta-Espar I. Weinstock L. D. Perez-Nievas B. G. Meltzer A. C. Siao Tick Chong M. Amaral A. C.. (2019). Distinct cytokine profiles in human brains resilient to Alzheimer's pathology. Neurobiol. Dis. 121, 327–337. 10.1016/j.nbd.2018.10.009 30336198 PMC6437670

[B2] Biron K. E. Dickstein D. L. Gopaul R. Jefferies W. A. (2011). Amyloid triggers extensive cerebral angiogenesis causing blood brain barrier permeability and hypervascularity in Alzheimer's disease. PLoS ONE 6:e23789. 10.1371/journal.pone.0023789 21909359 PMC3166122

[B3] Breiman L. (2001). Statistical modeling: the two cultures. Stat. Sci. 16, 199–231. 10.1214/ss/1009213726

[B4] Candelario-Jalil E. Yang Y. Rosenberg G. A. (2009). Diverse roles of matrix metalloproteinases and tissue inhibitors of metalloproteinases in neuroinflammation and cerebral ischemia. Neuroscience 158, 983–994. 10.1016/j.neuroscience.2008.06.025 18621108 PMC3584171

[B5] Craig-Schapiro R. Kuhn M. Xiong C. Pickering E. H. Liu J. Misko T. P.. (2011). Multiplexed immunoassay panel identifies novel CSF biomarkers for Alzheimer's disease diagnosis and prognosis. PLoS ONE 6:e18850. 10.1371/journal.pone.0018850 21526197 PMC3079734

[B6] Desai B. S. Schneider J. A. Li J. L. Carvey P. M. Hendey B. (2009). Evidence of angiogenic vessels in Alzheimer's disease. J Neural Transm. 116, 587–597. 10.1007/s00702-009-0226-9 19370387 PMC2753398

[B7] Elahi F. M. Casaletto K. B. La Joie R. Walters S. M. Harvey D. Wolf A.. (2020). Plasma biomarkers of astrocytic and neuronal dysfunction in early- and late-onset Alzheimer's disease. Alzheimers Dement. 16, 681–695. 10.1016/j.jalz.2019.09.004 31879236 PMC7138729

[B8] Iadecola C. (2013). The pathobiology of vascular dementia. Neuron 80, 844–866. 10.1016/j.neuron.2013.10.008 24267647 PMC3842016

[B9] Ishwaran H. Malley J. D. (2014). Synthetic learning machines. BioData Min. 7:28. 10.1186/s13040-014-0028-y 25614764 PMC4279689

[B10] Ito M. Komai K. Mise-Omata S. Iizuka-Koga M. Noguchi Y. Kondo T.. (2019). Brain regulatory T cells suppress astrogliosis and potentiate neurological recovery. Nature 565, 246–250. 10.1038/s41586-018-0824-5 30602786

[B11] Jack C. R. Jr. Bennett D. A. Blennow K. Carrillo M. C. Dunn B. Haeberlein S. B.. (2018). NIA-AA Research Framework: Toward a biological definition of Alzheimer's disease. Alzheimers Dement. 14, 535–562. 10.1016/j.jalz.2018.02.018 29653606 PMC5958625

[B12] Janelidze S. Zetterberg H. Mattsson N. Palmqvist S. Vanderstichele H. Lindberg O.. (2016). CSF Abeta42/Abeta40 and Abeta42/Abeta38 ratios: better diagnostic markers of Alzheimer disease. Ann. Clin. Transl. Neurol. 3, 154–165. 10.1002/acn3.274 27042676 PMC4774260

[B13] Jorgensen I. F. Aguayo-Orozco A. Lademann M. Brunak S. (2020). Age-stratified longitudinal study of Alzheimer's and vascular dementia patients. Alzheimers Dement. 16, 908–917. 10.1002/alz.12091 32342671 PMC7383608

[B14] Karanth S. Nelson P. T. Katsumata Y. Kryscio R. J. Schmitt F. A. Fardo D. W.. (2020). Prevalence and clinical phenotype of quadruple misfolded proteins in older adults. JAMA Neurol. 77, 1299–1307. 10.1001/jamaneurol.2020.1741 32568358 PMC7309572

[B15] Miao W. Zhao Y. Huang Y. Chen D. Luo C. Su W.. (2020). IL-13 ameliorates neuroinflammation and promotes functional recovery after traumatic brain injury. J. Immunol. 204, 1486–1498. 10.4049/jimmunol.1900909 32034062

[B16] Pantoni L. (2010). Cerebral small vessel disease: from pathogenesis and clinical characteristics to therapeutic challenges. Lancet Neurol. 9, 689–701. 10.1016/S1474-4422(10)70104-6 20610345

[B17] Pillai J. A. Maxwell S. Bena J. Bekris L. M. Rao S. M. Chance M.. (2019). Key inflammatory pathway activations in the MCI stage of Alzheimer's disease. Ann. Clin. Transl. Neurol. 6, 1248–1262. 10.1002/acn3.50827 31353852 PMC6649519

[B18] Rundhaug J. E. (2005). Matrix metalloproteinases and angiogenesis. J. Cell. Mol. Med. 9, 267–285. 10.1111/j.1582-4934.2005.tb00355.x 15963249 PMC6740080

[B19] Ryu J. K. Cho T. Choi H. B. Wang Y. T. McLarnon J. G. (2009). Microglial VEGF receptor response is an integral chemotactic component in Alzheimer's disease pathology. J. Neurosci. 29, 3–13. 10.1523/JNEUROSCI.2888-08.2009 19129379 PMC6664925

[B20] Schneider J. A. Arvanitakis Z. Bang W. Bennett D. A. (2007). Mixed brain pathologies account for most dementia cases in community-dwelling older persons. Neurology 69, 2197–2204. 10.1212/01.wnl.0000271090.28148.24 17568013

[B21] Snowdon D. A. Greiner L. H. Mortimer J. A. Riley K. P. Greiner P. A. Markesbery W. R. (1997). Brain infarction and the clinical expression of Alzheimer disease. The Nun Study [see comments]. JAMA 277, 813–817. 10.1001/jama.1997.03550020046024 9052711

[B22] Snyder H. M. Corriveau R. A. Craft S. Faber J. E. Greenberg S. M. Knopman D.. (2015). Vascular contributions to cognitive impairment and dementia including Alzheimer's disease. Alzheimers Dement 11, 710–717. 10.1016/j.jalz.2014.10.008 25510382 PMC4731036

[B23] Sonnen J. A. Santa Cruz K. Hemmy L. S. Woltjer R. Leverenz J. B. Montine K. S.. (2011). Ecology of the aging human brain. Arch. Neurol. 68, 1049–1056. 10.1001/archneurol.2011.157 21825242 PMC3218566

[B24] Stomrud E. Bjorkqvist M. Janciauskiene S. Minthon L. Hansson O. (2010). Alterations of matrix metalloproteinases in the healthy elderly with increased risk of prodromal Alzheimer's disease. Alzheimers Res. Ther. 2:20. 10.1186/alzrt44 20576109 PMC2919700

[B25] Tarkowski E. Issa R. Sjogren M. Wallin A. Blennow K. Tarkowski A.. (2002). Increased intrathecal levels of the angiogenic factors VEGF and TGF-beta in Alzheimer's disease and vascular dementia. Neurobiol. Aging 23, 237–243. 10.1016/S0197-4580(01)00285-8 11804709

[B26] Toledo J. B. Arnold S. E. Raible K. Brettschneider J. Xie S. X. Grossman M.. (2013). Contribution of cerebrovascular disease in autopsy confirmed neurodegenerative disease cases in the National Alzheimer's Coordinating Centre. Brain 136(Pt 9), 2697–2706. 10.1093/brain/awt188 23842566 PMC3858112

[B27] Tomimoto H. (2011). Subcortical vascular dementia. Neurosci. Res. 71, 193–199. 10.1016/j.neures.2011.07.1820 21821070

[B28] van Buuren S. Groothuis-Oudshoorn k. (2011). mice: multivariate imputation by chained equations in R. J. Stat. Softw. 45, 1–67. 10.18637/jss.v045.i03

[B29] Whelan C. D. Mattsson N. Nagle M. W. Vijayaraghavan S. Hyde C. Janelidze S.. (2019). Multiplex proteomics identifies novel CSF and plasma biomarkers of early Alzheimer's disease. Acta Neuropathol. Commun. 7:169. 10.1186/s40478-019-0795-2 31694701 PMC6836495

[B30] Winder Z. Sudduth T. L. Fardo D. Cheng Q. Goldstein L. B. Nelson P. T.. (2020). Hierarchical clustering analyses of plasma proteins in subjects with cardiovascular risk factors identify informative subsets based on differential levels of angiogenic and inflammatory biomarkers. Front. Neurosci. 14:84. 10.3389/fnins.2020.00084 32116527 PMC7016016

