# Supplementary material for: Inflammatory Biomarkers Aid in Diagnosis of Dementia
Source: Front Aging Neurosci. 2021 Aug 17;13:717344. doi: 10.3389/fnagi.2021.717344 (PMC8416621; doi:10.3389/fnagi.2021.717344)
Supplement: Supplementary file 1 [file Data_Sheet_1.docx]

# Supplemental Material

## Univariate plots


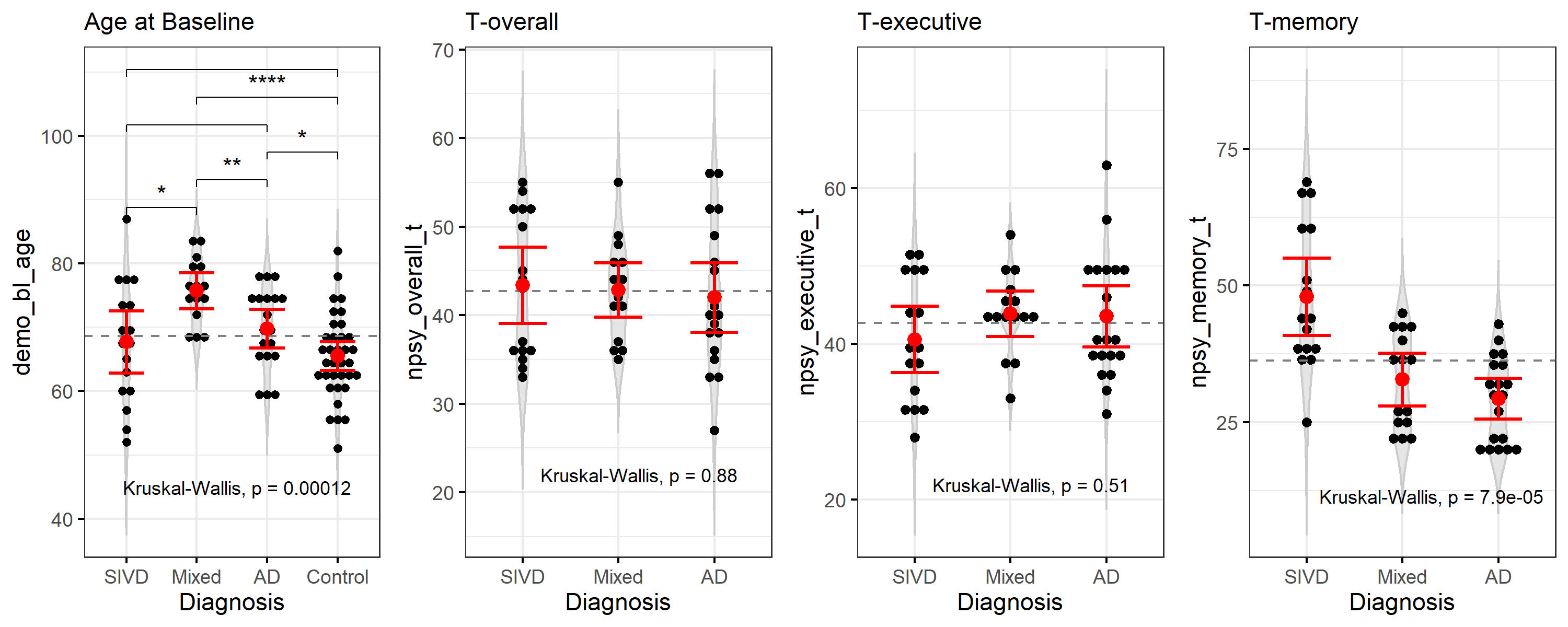


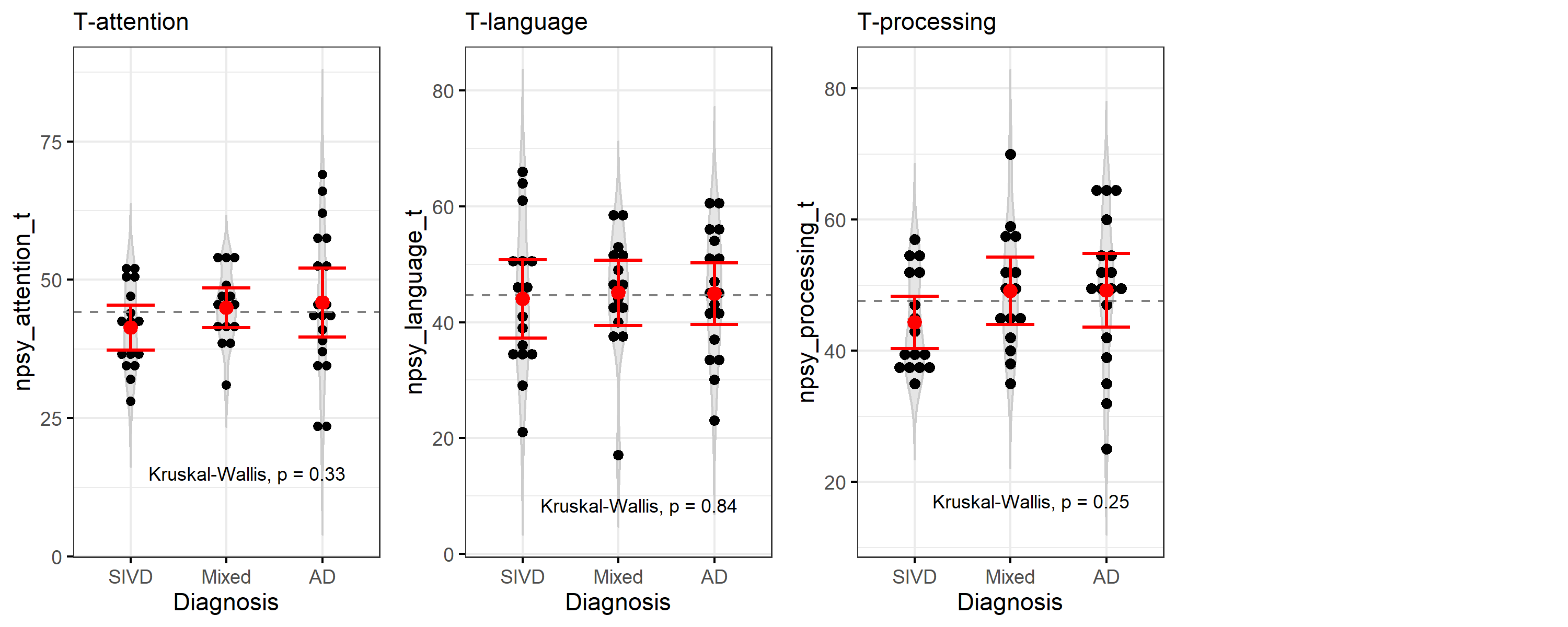


Supplement Figure 1. Univariate plots of age and neuropsychological test feature values by diagnosis for individual patients, means with 95% confidence intervals, and pairwise nonparametric comparisons of the medians between all four diagnosis groups.


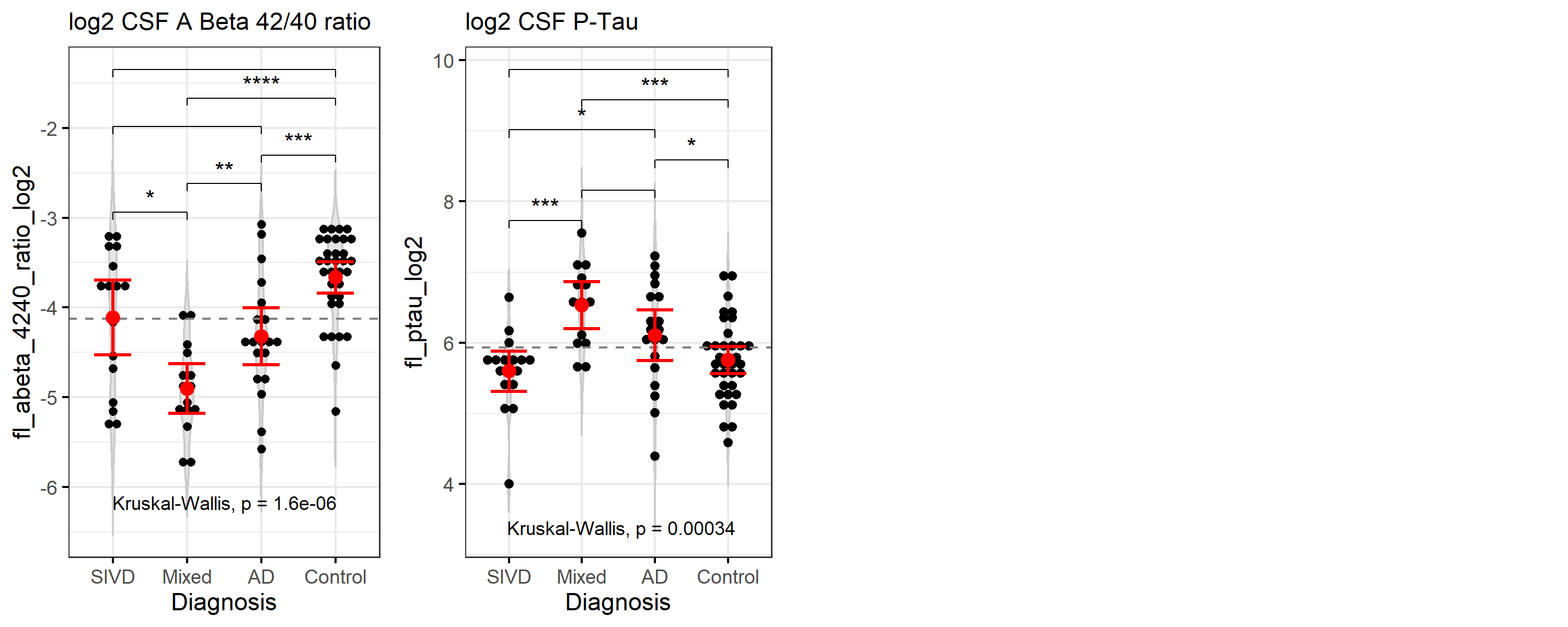


Supplement Figure 2. Univariate plots of Alzheimer’s disease protein feature values by diagnosis for individual patients, means with 95% confidence intervals, and pairwise nonparametric comparisons of the medians between all four diagnosis groups.


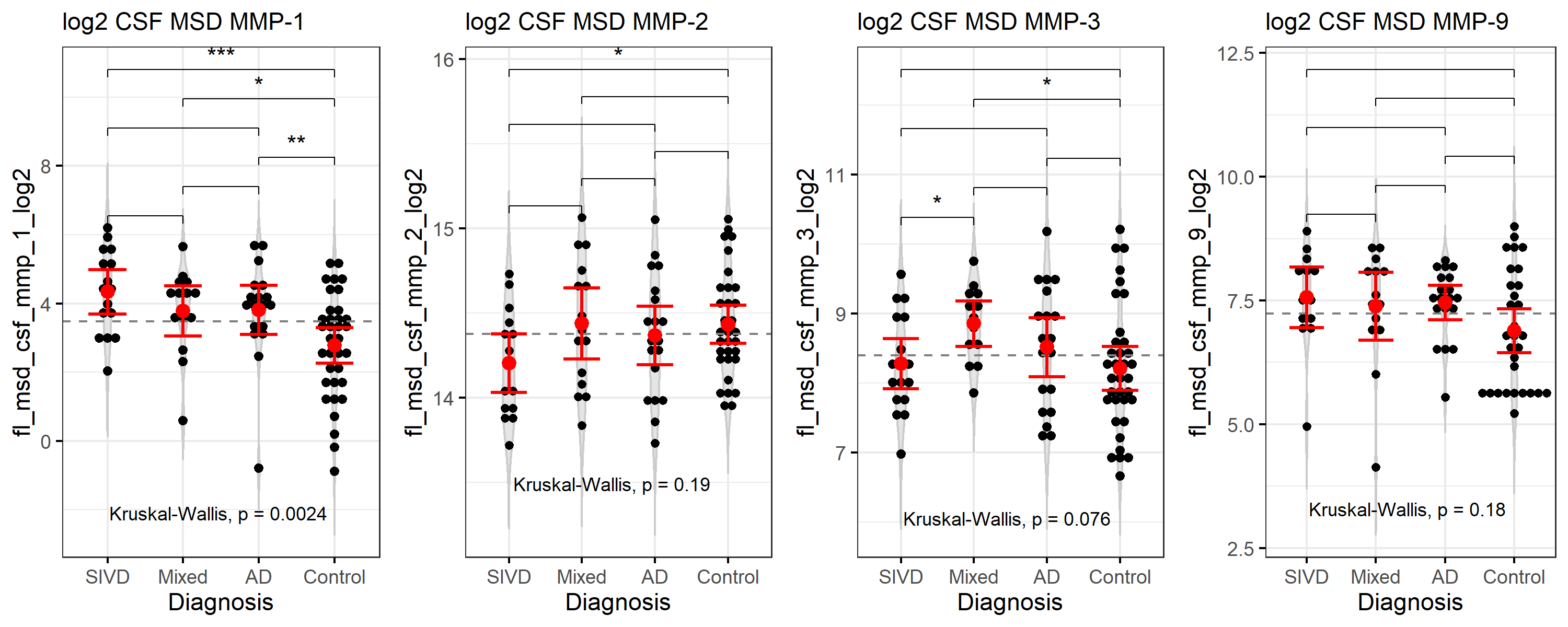


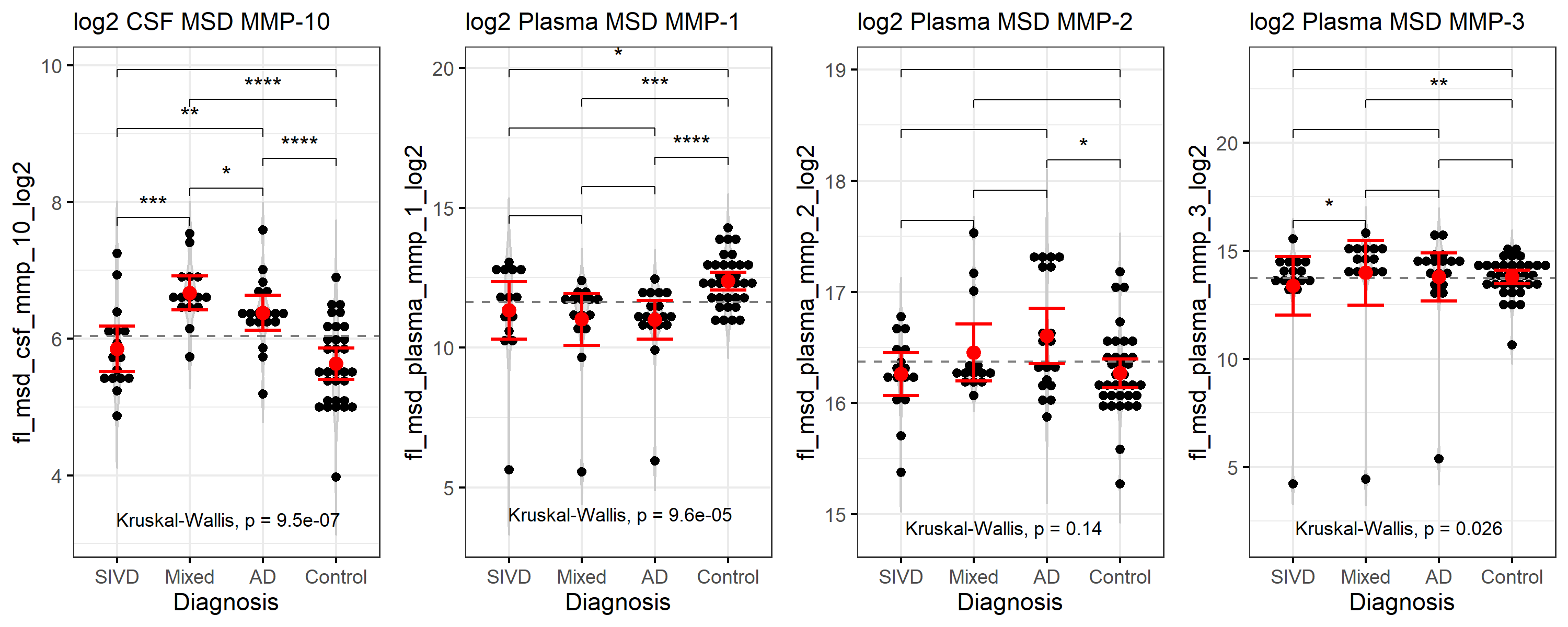


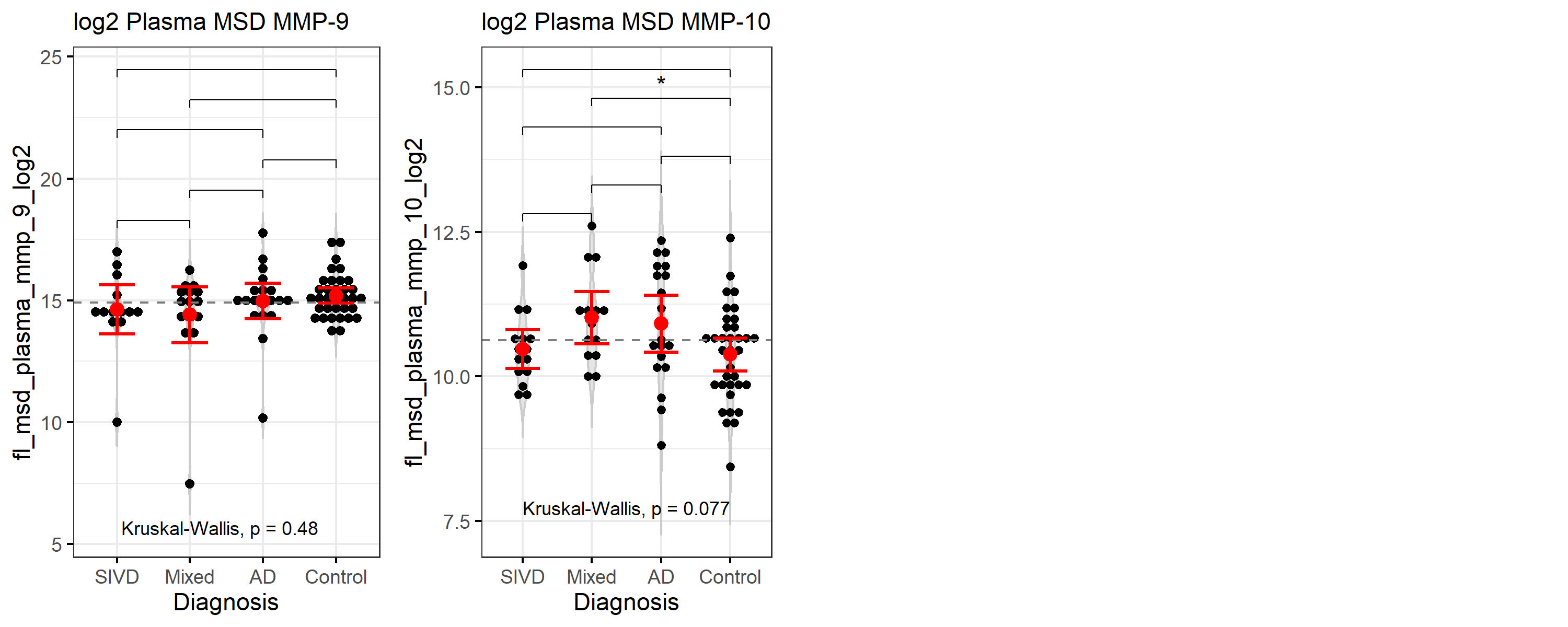


Supplemental Figure 3. Univariate plots of Protease feature values by diagnosis for individual patients, means with 95% confidence intervals, and pairwise nonparametric comparisons of the medians between all four diagnosis groups.


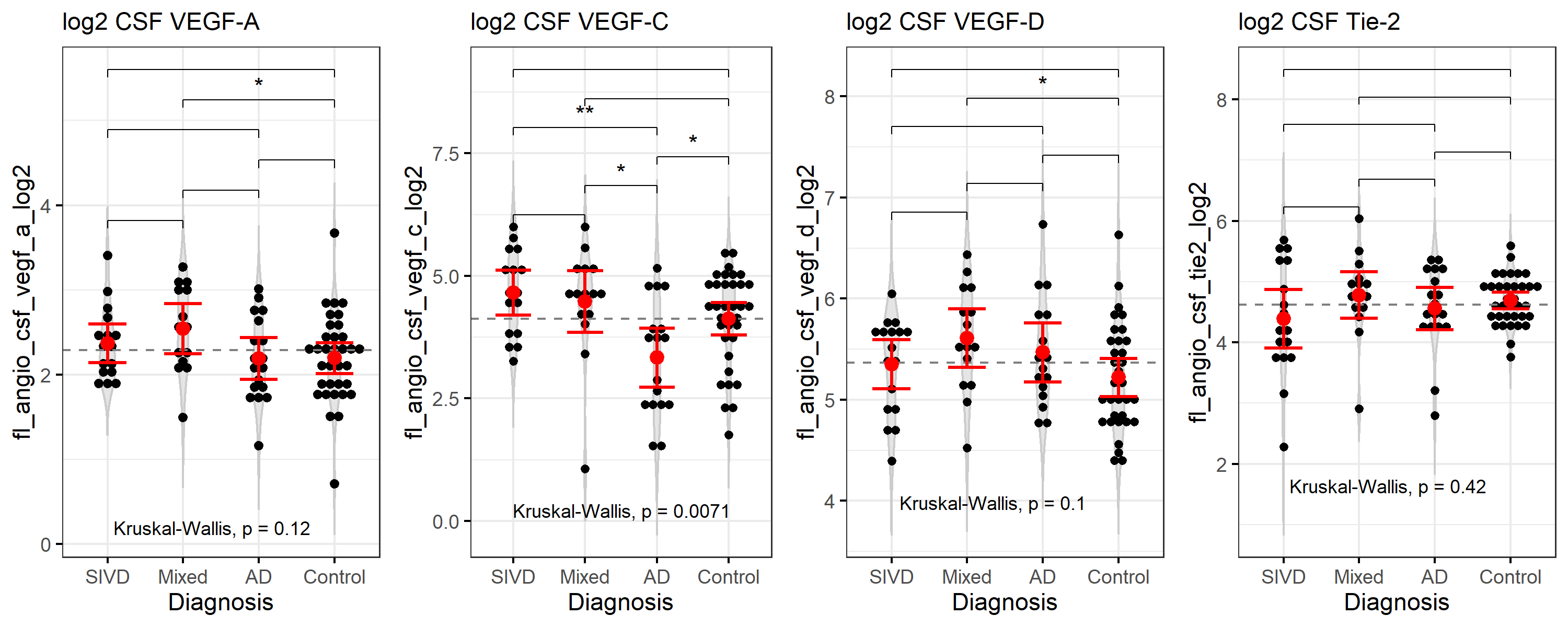


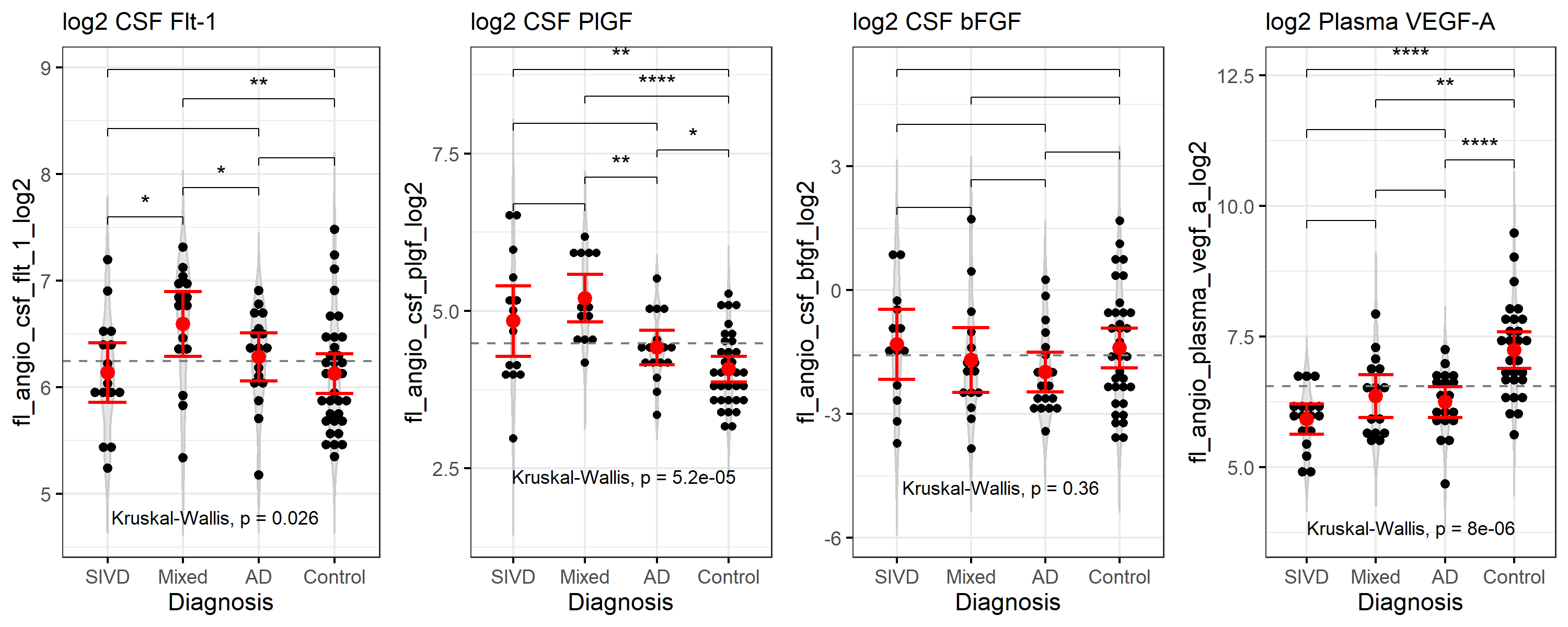


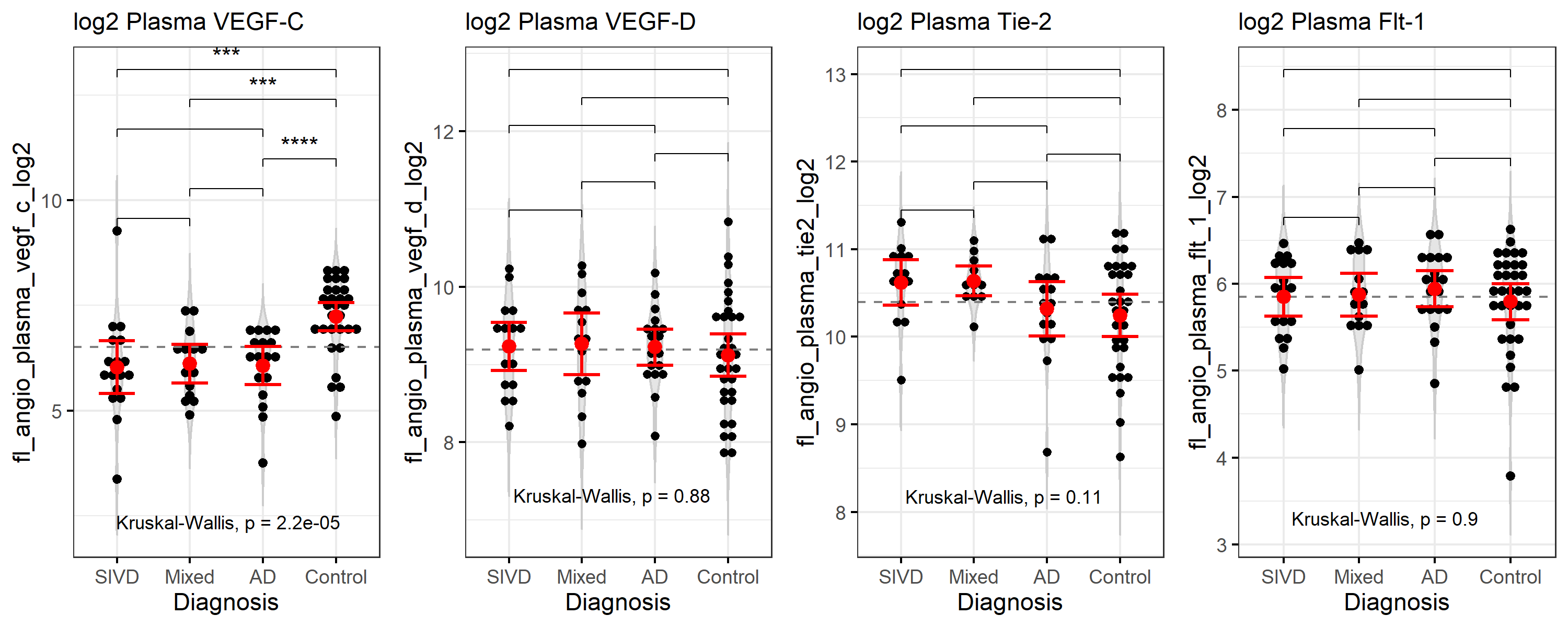


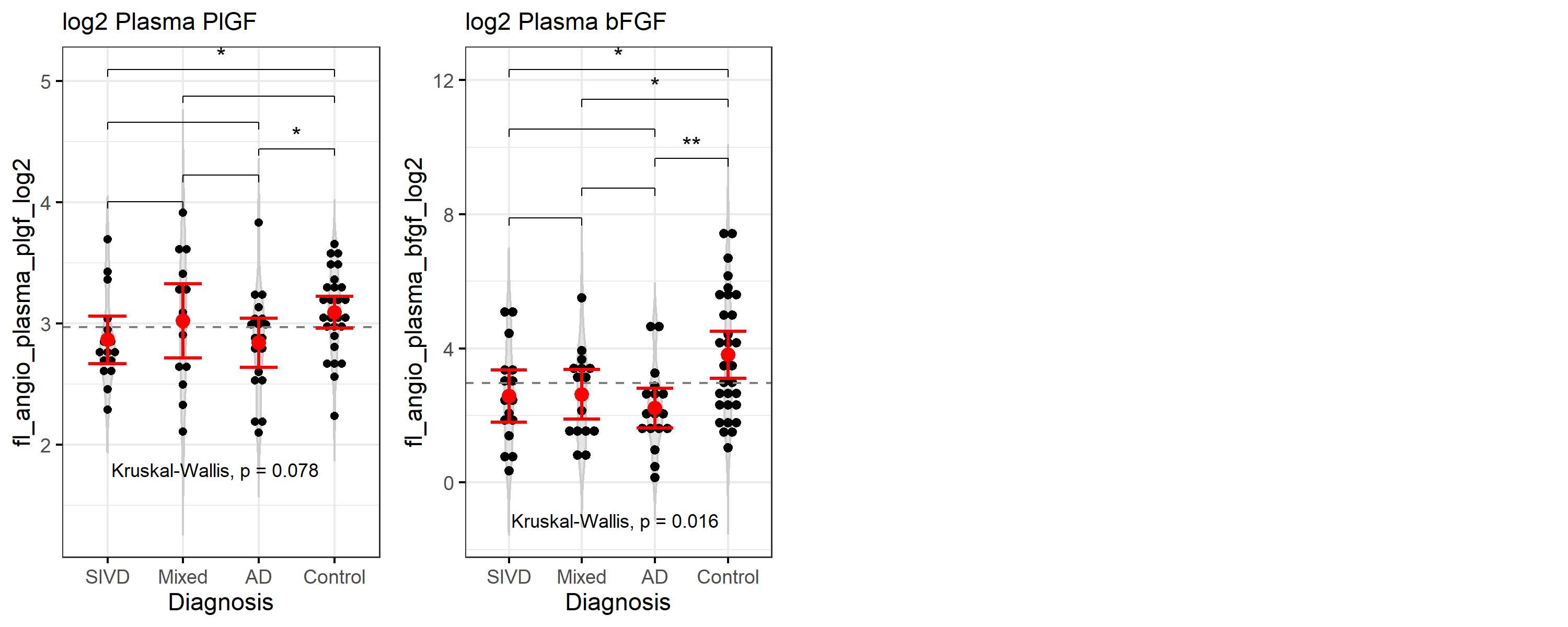


Supplemental Figure 4. Univariate plots of Angiogenesis feature values by diagnosis for individual patients, means with 95% confidence intervals, and pairwise nonparametric comparisons of the medians between all four diagnosis groups.


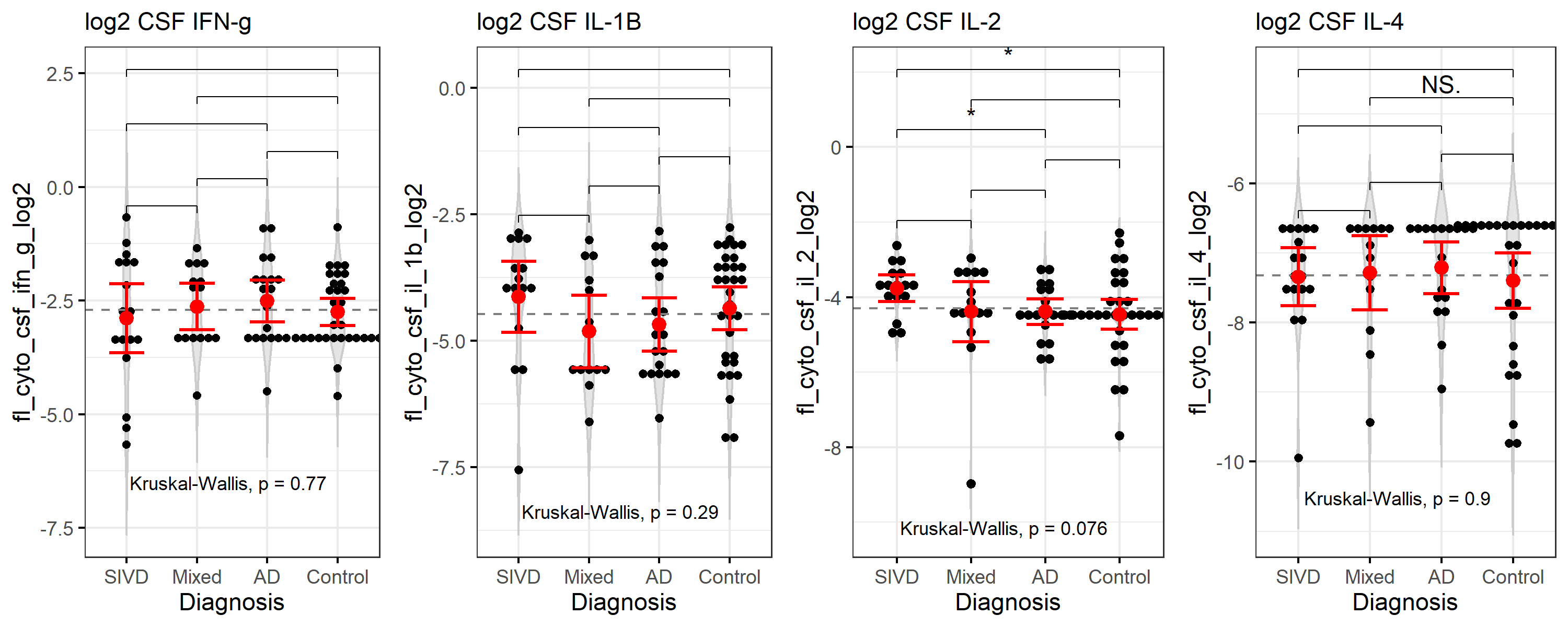


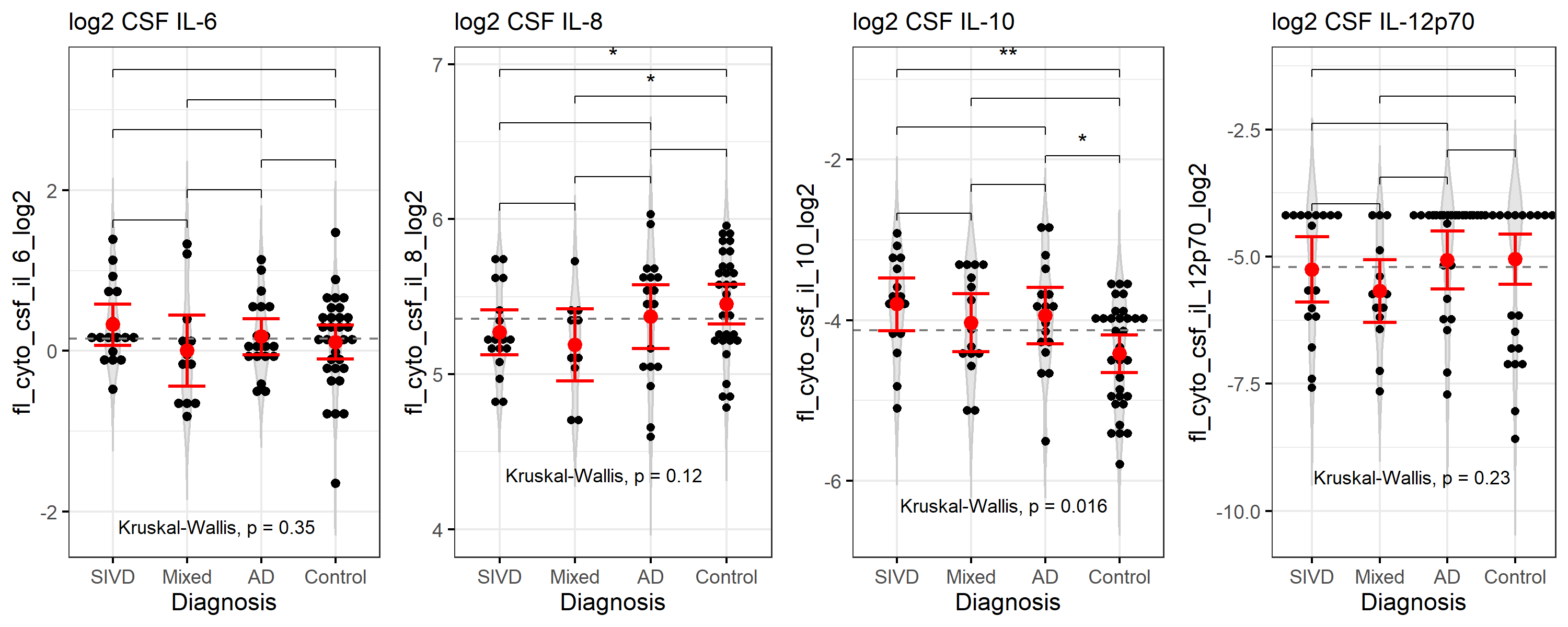


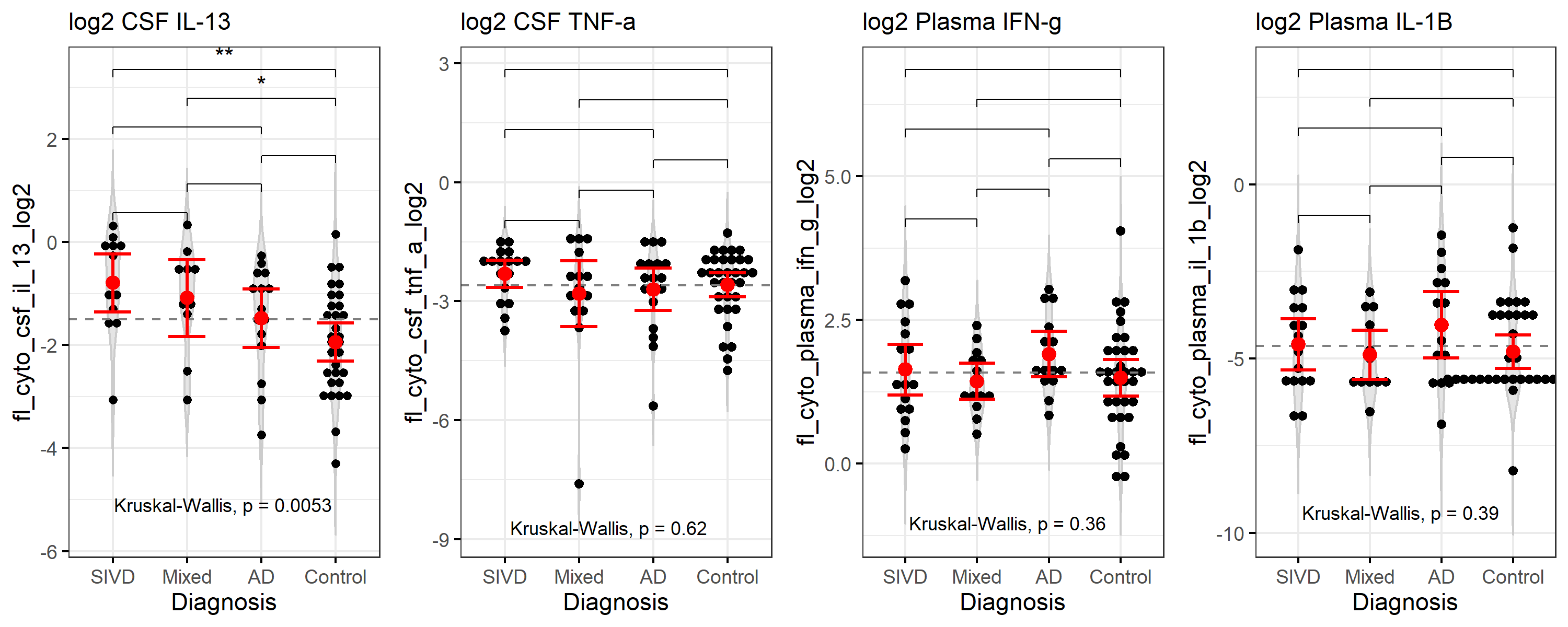


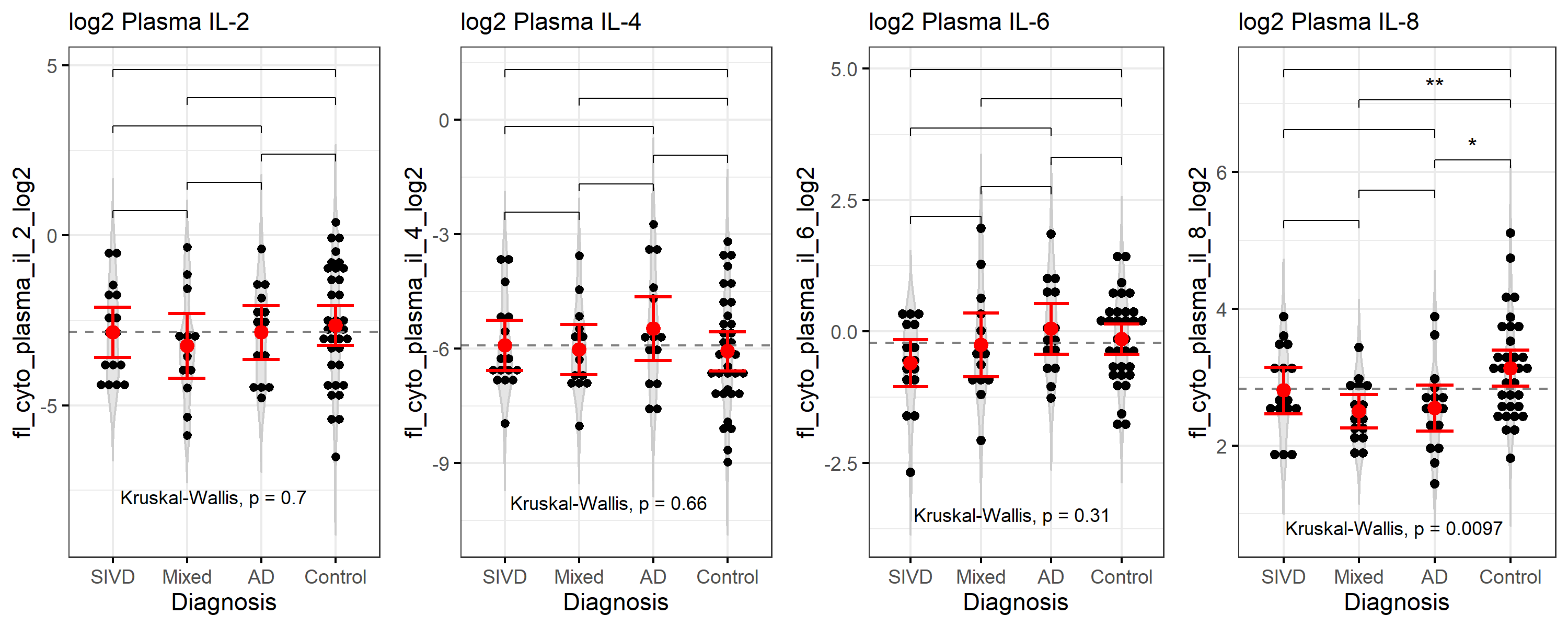


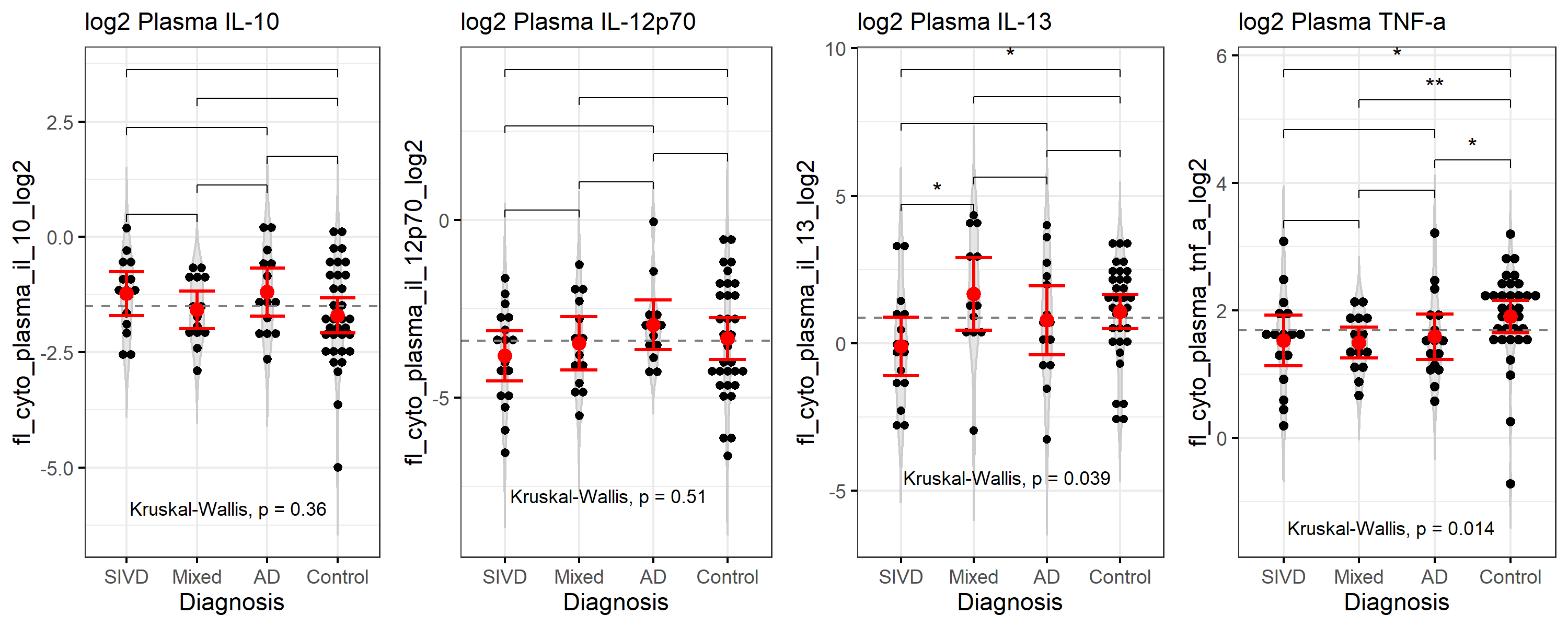


Supplemental Figure 5. Univariate plots of Cytokine feature values by diagnosis for individual patients, means with 95% confidence intervals, and pairwise nonparametric comparisons of the medians between all four diagnosis groups.
